# Seroconversion and Kinetics of Vibriocidal Antibodies during the First 90 Days of Re-Vaccination with Oral Cholera Vaccine in an Endemic Population

**DOI:** 10.3390/vaccines12040390

**Published:** 2024-04-08

**Authors:** Caroline Cleopatra Chisenga, Bernard Phiri, Harriet Ng’ombe, Mutinta Muchimba, Kalo Musukuma-Chifulo, Suwilanji Silwamba, Natasha Makabilo Laban, Chaluma Luchen, Fraser Liswaniso, Kennedy Chibesa, Cynthia Mubanga, Kapambwe Mwape, Michelo Simuyandi, Adam F. Cunningham, David Sack, Samuel Bosomprah

**Affiliations:** 1 Enteric Disease and Vaccine Research Unit, Centre for Infectious Disease Research in Zambia, Lusaka P.O. Box 34681, Zambia; caroline.chisenga@cidrz.org (C.C.C.); bernard.phiri@cidrz.org (B.P.); harriet.ngombe@cidrz.org (H.N.); mutinta.muchimba@cidrz.org (M.M.); kalo.musukuma@cidrz.org (K.M.-C.); suwilanji.silwamba@cidrz.org (S.S.); natasha.laban@cidrz.org (N.M.L.); chaluma.luchen@cidrz.org (C.L.); fraserliswaniso@gmail.com (F.L.); kennedy.chibesa@cidrz.org (K.C.); cynthia.mubanga@cidrz.org (C.M.); kapambwe.mwape@cidrz.org (K.M.); michelo.simuyandi@cidrz.org (M.S.); 2 Institute of Immunology and Immunotherapy, University of Birmingham, Edgbaston, Birmingham B15 2TT, UK; a.f.cunningham@bham.ac.uk; 3 Center for Immunization Research, Johns Hopkins Bloomberg School of Public Health, Baltimore, MD 21205, USA; dsack1@jhu.edu; 4 Department of Biostatistics, School of Public Health, University of Ghana, Accra P.O. Box LG13, Ghana

**Keywords:** vibrio cholerae, Shanchol vaccine, immunogenicity, HIV, waning, cholera priority areas

## Abstract

Despite the successful introduction of oral cholera vaccines, Zambia continues to experience multiple, sporadic, and protracted cholera outbreaks in various parts of the country. While vaccines have been useful in staying the cholera outbreaks, the ideal window for re-vaccinating individuals resident in cholera hotspot areas remains unclear. Using a prospective cohort study design, 225 individuals were enrolled and re-vaccinated with two doses of Shanchol™, regardless of previous vaccination, and followed-up for 90 days. Bloods were collected at baseline before re-vaccination, at day 14 prior to second dosing, and subsequently on days 28, 60, and 90. Vibriocidal assay was performed on samples collected at all five time points. Our results showed that anti-LPS and vibriocidal antibody titers increased at day 14 after re-vaccination and decreased gradually at 28, 60, and 90 days across all the groups. Seroconversion rates were generally comparable in all treatment arms. We therefore conclude that vibriocidal antibody titers generated in response to re-vaccination still wane quickly, irrespective of previous vaccination status. However, despite the observed decline, the levels of vibriocidal antibodies remained elevated over baseline values across all groups, an important aspect for Zambia where there is no empirical evidence as to the ideal time for re-vaccination.

## 1. Introduction

Though the rolling out of the oral cholera vaccine (OCV) Shanchol™ has been successful and is a positive step for the Zambian government, with evidence of it being immunogenic among the vaccinated populations [1], Zambia continues to record cholera cases that are now extending to areas previously not known to be cholera hotpots. The spreading, recurrent, and protracted cholera outbreaks which are being experienced in the era of reactive vaccination campaigns are of concern, especially with the World Health Organisation (WHO) set target of ending cholera by 2030. 

In 2016, Shanchol™ was deployed to Zambia for the first-time targeting individuals within hotspot areas to mitigate the outbreak. Thousands of individuals were vaccinated, the majority with only one dose. In 2020, another stock of OCV was received and given to individuals in identified hotspot areas regardless of prior vaccination status. Also, to stay the spread of cholera, recently (2022/2023) more vaccines have been distributed in areas recording cholera cases, especially those bordering Malawi, Congo DR, and Mozambique.

Post the introduction of OCVs, studies have been conducted in cholera hotspot areas to assess its immunogenicity [1], delayed second dose regimen [2], its effect during HIV infection [3], and the effect of ABO blood types on OCV immunogenicity in vaccinated individuals [4]. However, there is limited information on the antibody kinetics in the first three months after re-vaccinating individuals resident in cholera hotspot areas. This information is urgently needed, especially considering that Zambia’s neighbouring countries, i.e., Malawi, Tanzania, Zimbabwe, Mozambique, and Congo DR, continue to report cholera outbreaks [5]. Also, Zambia remains vulnerable to importing *Vibrio cholerae* because of ongoing trade in boarder areas coupled with them being porous. This is likely to contribute to significant delays in achieving the goal of ending cholera. Additionally, with global OCV shortages being experienced, there is an urgent need for each country reporting cholera outbreaks to be strategic on how vaccines are to be deployed, more-so with the recent introduction of the OCV single-dosing strategy. For instance, while a single-dose regimen has been employed in Zambia, the duration of protection remains questionable, with the high environmental enteric dysfunction (EED) [6] and HIV burden that can affect oral vaccine uptake [3].

We imagine that the distribution of vaccines in a reactive manner can potentially contribute to prolonged cholera outbreaks, as these are likely not targeting the individuals that may need them the most. Thus, the overarching aim for this study was to determine which individuals to prioritise for revaccinations for a sustained immunity against cholera among vulnerable populations. This study estimates the effect of revaccination on seroconversion, and characterizes the kinetics of vibriocidal antibodies. Revaccination is defined as having been previously fully vaccinated (received two doses of OCV) or partially vaccinated (received a single dose only) compared to naïve individuals (never been previously vaccinated).

## 2. Materials and Methods

### 2.1. Study Design

**Figure 1:**
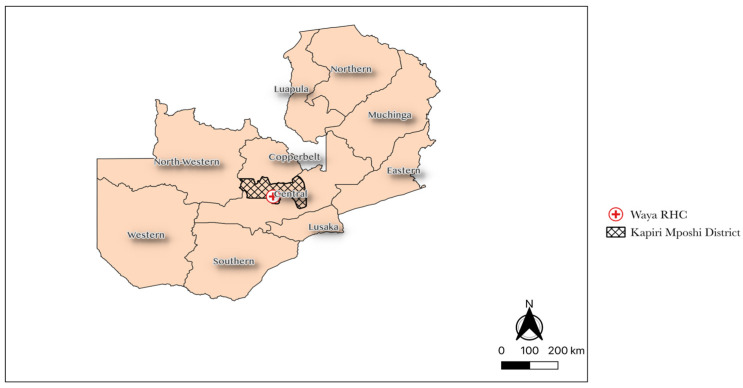
Shows the location for Waya Rural Health Centre.

This was a 3-parallel group observational cohort study conducted in the Lukanga Swamps, a cholera hotspot area in Central Province where over 10,000 OCVs were deployed in 2016; see Figure 1. The study was conducted between October 2021 and October 2022.

### 2.2. Recruitment and Enrolment

We held in-person sensitisation meetings with community stakeholders to inform them about the study. We hired and trained a total of 20 community volunteers on all study activities. Following training, community volunteers went around the villages engaging and reminding previously vaccinated individuals to make their way to the clinic if they were interested in learning more and or in being recruited in the OCV vaccination campaign. Interested individuals were given more information (study information sheet) on the study in the language most comfortable to them (either Bemba, Nyanja, or English) by the study staff. If willing to participate in the study, an informed consent form was signed. 

We enrolled participants if they were aged between 18 to 65 years and were residents of Lukanga Swamps. All participants provided written informed consent. Participants were excluded if they were participating in a similar study, had recently received OCV, had diarrhoea in the last 7 days, were on antibiotic treatment, or were pregnant. Participant groups were defined based on their previous vaccination status, namely, naïve (i.e., not previously vaccinated with OCV), partial dose (i.e., previously vaccinated with 1 dose OCV), and full dose (i.e., previously vaccinated with 2 doses OCV). 

### 2.3. Study Procedures

Each group of participants were given two doses of Shanchol™ (Shantha Biotechnics Private Limited, Hyderabad, India), per the manufacturer’s recommendation, i.e., 14 days apart. Participants were monitored for any adverse events following vaccination for 30 min, after which they were allowed to go home. Upon release, participants were encouraged to return to the study site or to contact any study staff if they felt unwell after receipt of the vaccine. All the vaccines were kept at 2–8 °C on site, as recommended by the manufacturers.

**Figure 2:**
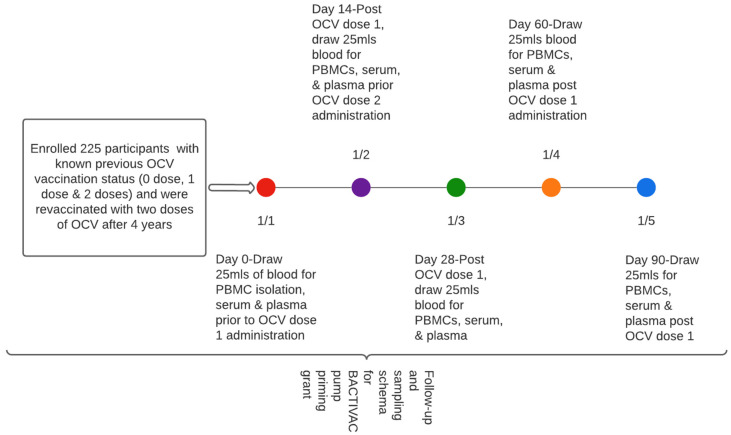
Participant sampling schema.

Approximately 20 mls of blood was collected at baseline (before vaccination with initial dose) at day 14 prior to receiving the second dose and subsequently at days 28, 60, and 90, respectively (Figure 2).

### 2.4. Laboratory Procedures

Vibriocidal antibody assay: We measured vibriocidal antibody titers at all five time points using the guinea pig complement and *V*. *cholerae* O1 Ogawa PIC158 and Inaba PIC018 as the target organisms, as previously described [7]. Monoclonal antibodies that bind to the O-specific polysaccharide (OSP) moiety of LPS were used as a standard to monitor intra-assay variability between plates [8]. Briefly, colonies from overnight cultures were inoculated in Brain Heart Infusion (BHI) broth and incubated at 37 °C for about 4 h before harvesting the cells. Heat-inactivated serum, exogenous guinea pig complement (Sigma Aldrich S1639-5ML, Dallas, TX, USA), and *V*. *cholerae* bacterial cells were then put in 96-well microtitre tissue culture plates (Life sciences, Durham, NC, USA) and incubated at 37 °C. Vibriocidal titres were defined as the 595 nm reciprocal of the highest serum dilution, resulting in a 50% reduction in optical density read compared to positive control wells without serum. Seroconversion was defined as a 4-fold or greater increase from the baseline vibriocidal titres [9].

**LPS ELISAs:** A standardized enzyme-linked immunosorbent assay (ELISA) was employed to quantify plasma IgA, IgG, and IgM antibody responses against V. cholerae lipopolysaccharide (LPS), following established protocols [10,11]. ELISA plates (Nunc, low affinity) were coated with 2.5 μg/mL LPS, prepared in carbonate buffer. Subsequently, the plates were incubated with 100 μL of plasma (diluted at 1:50). The bound antibodies were then probed using horseradish peroxidase-conjugated goat anti-human IgA, IgG, and IgM antibodies (Jackson Immuno Laboratories, Baltimore, MD, USA). The colorimetric substrate ABTS/H_2_O_2_ (Sigma-Aldrich, St. Louis, MO, USA) was employed, and absorbance values were measured at a wavelength of 405 nm using kinetic readings (milliabsorbance/second).

### 2.5. Statistical Analyses

Sample size was calculated based on a Cochran–Armitage test for trend in proportions [12], and sample sizes of 54, 54, and 54 were obtained from 3 groups with equally spaced doses (0, 1 and 2) and proportions equal to 0.05, 0.15, and 0.25, respectively. The total sample of 162 participants achieved 80% power to detect a linear trend using a two-sided Z test with continuity correction and a significance level of 0.05. Sample size calculation was done using PASS 16.0.

Background characteristics were summarized using frequency and proportion for categorical variables, while median and interquartile interval were used for continuous variables. Previous exposure to vibriocidal cholerae was defined as IgM titre greater than 80. We compared trends using a chi-square test for trend. Logistic regression was used to determine the influence of previous vaccination status on seroconversion, adjusting for key confounding variables. Seroconversion was defined as a four-fold rise in vibriocidal geometric mean titres from baseline titres [13]. We set statistical significance at alpha < 0.05, and all statistical analyses were performed using Stata 16 MP (StataCorp, College Station, TX, USA).

### 2.6. Ethical Statement

Ethical approval was obtained from the University of Zambia Biomedical Research Ethics Committee (UNZABREC) reference number 007-12-16, while the National Health Research Authority provided the authorization to conduct the study. Written informed consent was also obtained from all enrolled participants into the study. All study procedures were conducted according to good clinical practice guidelines and by trained medical personnel.

## 3. Results

**Figure 3:**
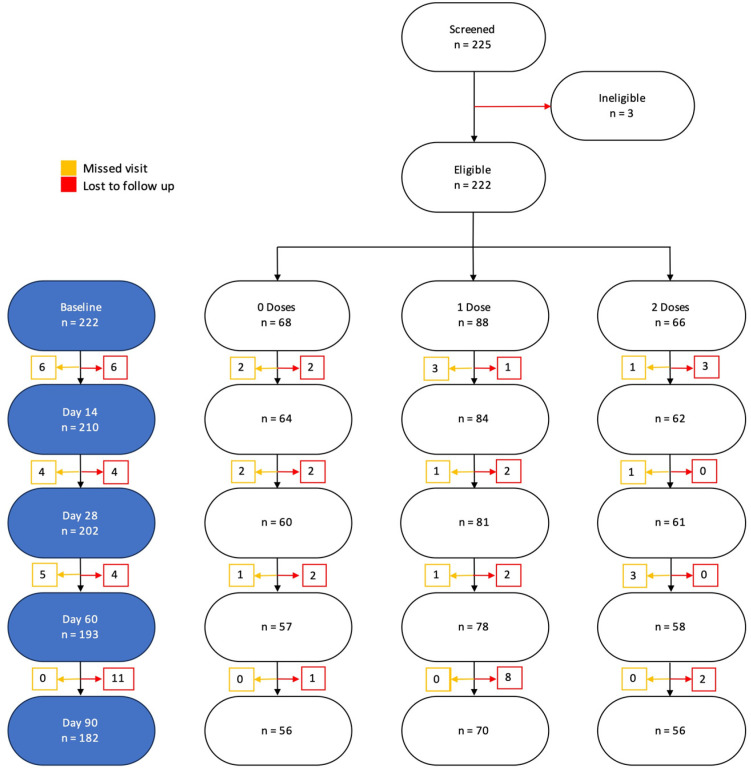
Participant CONSORT diagram and final number included in the analysis and reasons for exclusion.

A total of 225 participants were enrolled into the study. Of these, we excluded 40 who did not follow-up and 3 who were missing some baseline characteristics. Overall, 182 were included in the final analysis (Figure 3).

### 3.1. Baseline Characteristics of the Enrolled Participants

Among the 182 participants included in the final analysis, 56 (31%) had previously not received any vaccination (vaccine naïve), 70 (38%) were previously vaccinated with a single dose of OCV, and another 56 (31%) had received two doses. Furthermore, 58% were females, 63% were HIV-negative, and 37% were aged between 26–45 years old. Per the Fisher’s exact test, we recorded significant differences across treatment arms on gender (*p =* 0.003) and age (*p* = 0.002) (Table 1).

### 3.2. Kinetics of Vibriocidal Antibody Titre 90 Days after Re-Vaccination

**Figure 4:**
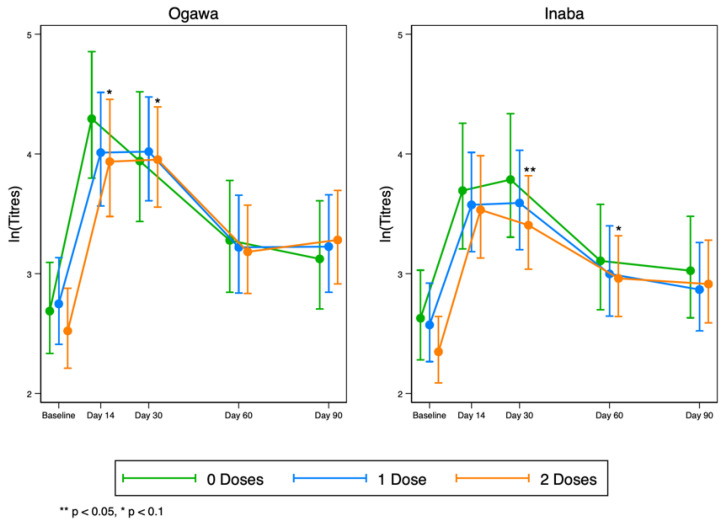
Kinetics of serum vibriocidal geometric mean titres to LPS at five time points before and after OCV re-vaccinations to Ogawa (**left**) and Inaba (**right**) cholera serotypes.

Overall, we observed a rise in vibriocidal antibody titres to LPS in all groups by day 14, which then began to wane after day 30 for both Ogawa and Inaba (Figure 4). These results suggest that any pre-existing responses to the vaccine in the original vaccination programme do not necessarily interfere with the responses after the vaccine was re-introduced.

At day 30, after full dosing, individuals who previously had a double dose were about 26% more likely to seroconvert compared to the naïve group; however, this protective effect is likely to be due to chance (Adjusted risk ratio (aRR) = 1.26; 95%CI: 0.86, 1.84; *p* = 0.237) (Table 2). On the other hand, on day 15, after a single dose was given, results show that individuals who previously had a single or double dose were about 29% less likely to seroconvert compared to the naïve group (aRR = 0.69; 95%CI: 0.50, 0.93; *p* = 0.017) (Table 2).

**Figure 5:**
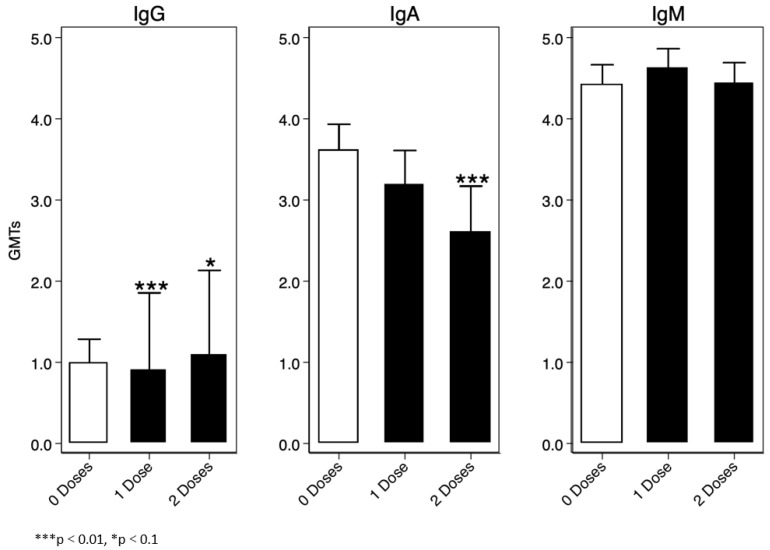
Baseline IgG, IgA, and IgM titres.

For the immunoglobulin analysis, we assumed IgG, IgA, and IgM values were a mixture of two lognormal distributions, and consequently we used a Finite Mixture Model (FMM) to determine a threshold for sero-prevalence. One of the two distributions represented sero-negative participants, and the other represented sero-positive participants. We defined the cut-off as the mean log10(IgX) (IgX = collective name) of the log-normal distribution of the sero-negative population plus three standard deviations. Overall, we found that IgM was the highest immunoglobulin at baseline amongst the enrolled participants, the lowest being IgG Figure 5.

In the IgG positive group, vibriocidal geometric mean antibody titres to Ogawa were generally slightly higher in those who were naïve or had previously received two doses of OCV compared to those who were vaccinated with a single dose (Supplementary Figure S1).

Similarly, vibriocidal geometric mean antibody titres to Inaba serotype were generally slightly higher in those who were naïve or had previously received two doses of OCV compared to those who were vaccinated with a single dose (Supplementary Figure S2).

Participants categorised as IgA-positive and who were previously given two doses of OCV or were vaccine-naïve had high vibriocidal geometric mean antibody titres Ogawa serotype compared to individuals previously vaccinated with a single dose (Supplementary Figure S3). This was also the case for Inaba serotype, as shown in Supplementary Figure S4 below.

Participants categorised as IgM-positive and or negative had a similar trend in vibriocidal geometric mean antibody titres to both serotypes Inaba and Ogawa (Supplementary Figures S5 and S6).

Furthermore, grouping all participants showed that HIV-positive individuals were generally trending lower than their counterparts to both serotypes (Ogawa and Inaba) on all time points, as can be seen in Supplementary Figures S7 and S8.

## 4. Discussion

This study sought to investigate the seroconversion and kinetics of vibriocidal antibody titres in three groups of individuals, i.e., naïve, or vaccinated with one or two doses of oral cholera vaccine 4 years back, to determine which individuals to prioritise for revaccination among residents in cholera hotspot areas of Zambia.

Overall, revaccination after 4 years was not superior over the naïve population resident in cholera hotspot areas. The seroconversion rates among the studied groups were also similar, even though the adults were more likely to seroconvert when compared to the younger ones. In addition, the vibriocidal antibody kinetics during the first 90 days of vaccinations was similar among all the treatment arms. This trend was not affected by (i) serotype (Ogawa or Inaba), (ii) previous or recent exposure to cholera when immunoglobulins (IgA, IgM, and IgG) were investigated with IgM being the most circulating immunoglobulin in the population, and that (iii) vibriocidal antibodies were a little lower in persons living with HIV when compared to their counterparts.

Generally, vibriocidal antibody titers remain an indirect surrogate marker for longer-term immunity [14,15]. And in Zambia, the recently established vibriocidal assay is one of the reliable assays that is being used to measure cholera-specific antibodies in different scenarios [1,2,3,4]. Undoubtably, for the assessment of mucosal immunity, they certainly are a good proxy [16]. While vibriocidal antibody titers have shown reliability as a correlate of protection against cholera, they still are an imperfect marker for long-term immunity, as can be seen by the quick drop in our population. Particularly, a recent review has documented that there is no threshold for complete protection with the vibriocidal antibody titers [17]. Thus, the correlation of vibriocidal antibody titers with protection remains debatable.

In our quest of understanding which groups of individuals to prioritise for revaccinations in cholera hotspot areas, i.e., vaccine naïve versus revaccinated individuals, we found no significant difference in vibriocidal kinetics in all groups during the first 90 days post vaccinations. In addition, we also noted that naïve individuals had slightly elevated vibriocidal geometric mean titres on all measured time points compared to the revaccinated individuals, which is contrary to what is reported by Chowdhury and colleagues [18]. We postulated that the lack of vaccine boosting effect, noted in our population when compared to what Chowdhury et al. reported in their study, could possibly be explained by the longer wait (4 years in our case, compared to the 3-year wait before booting. However, in both studies there was an observed quick rise and fall of vibriocidal antibodies after vaccination. These findings are consistent with our previous report in Zambia [1].

Furthermore, with the recent shift to the single-dose strategy for cholera outbreak control, our results demonstrate that a single dose may not be protective in the first 90 days after vaccination when the risk ratio was calculated. With the current ongoing cholera outbreak in Zambia in over 40 districts, where cases cumulatively are now standing at slightly over 7000 since the outbreak was reported, it is likely that the rise in cases might be due to the quick waning of the vaccine when a single dose is given, especially considering that some districts like Lusaka have received more than three single-dose vaccination campaigns in a space of 5 years. Thus, with our risk ratio showing that full dosing is protective in the first 90 days post vaccination, we imagine that a single dose might not be sufficient for controlling cholera in the first 90 days during an epidemic. Also, we found that vaccine immune response was not affected by serotype in our population, similar to what has been reported elsewhere [19]. Seroconversion rates were equally comparable in all groups.

We also assessed whether previous (IgG and IgM) exposure or current infection (IgA) to cholera enhances immunogenicity after re-vaccination, and found that, generally, IgG titres were significantly higher in those who had previously received a single or two doses of OCV compared to those who were unvaccinated at the time. Because the IgG antibodies have been found to persist for a prolonged period and penetrate the lumen of the intestine more efficiently than the IgM, it is hypothesised that this may lead to a booting effect and ultimately to a longer-lasting protection, as shown in one study [20]. However, in our study, we observed that despite high baseline IgG and or IgM, the immunogenicity to the vaccine in all groups was basically the same. Other reports also show that the presence of IgG can mediate protection through inhibition of intestinal adherence and colonisation activities of vibrios [20]. For IgA antibody titres, we found that individuals who did not receive any vaccine were likely to have recent infection with cholera followed by those vaccinated with a single dose only than those who received two doses. The presence of IgA antibodies indicates recent infection. As such, being fully vaccinated appeared to be protective, as these groups of individuals had significantly lower IgA antibody titres. It may also mean that there is sustained memory after priming the body with two doses of OCV in fully vaccinated individuals. However, in our case, we did not specifically test for secretory IgA, and thus cannot confirm these findings [21].

Vibriocidal activity in both naïve and revaccinated adults living with HIV was also assessed, and found that, similar to the results previously reported [3], persons living with HIV had slightly lower titres when compared to their counterparts. These findings were similar to other studies [21].

This study demonstrates that, (i) though the antibodies wane quickly and do not return to baseline levels, revaccination campaigns could be considered after 30 days, resources allowing; (ii) previous exposure does not give any booting, as the antibody titre levels were comparable in all study groups; (iii) persons living with HIV generally have lower vibriocidal antibodies at baseline, which could indicate a less robust immune response post-vaccination; and (iv) there might be poor memory of the oral cholera vaccine, seeing that response to revaccination was not superior to the naïve group.

The limitations are that, (i) even though we collected peripheral blood mononuclear cells for assessment of memory B cells at all time points, we lost viability due to liquid nitrogen shortages; (ii) we could not follow-up the participants to track vibriocidal antibody titres over time; (iii) we did not include the younger children (15 years and below) who are more vulnerable; and (iv) we could not specifically test for mucosal immune response using the secretory IgA.

Our study also provides guidance for policy to explore the use of alternative vaccines with booster schedules, such as Euvichol, for a sustained protection against cholera. Furthermore, if the current vaccine in use is waning quickly in adults, there is an urgent need to explore vaccine performance in Zambian under-five children who are more vulnerable and have previously shown poor immunogenicity after vaccination [2] and might be challenged with environmental enteropathy.

## 5. Conclusions

This study highlights the quick wanning of vibriocidal antibodies even in revaccinated individuals. However, while other researchers have reported that wanning does not mean loss of protection, there is need to urgently explore this quick wanning using novel technologies such as the systems serology in our population.

## Figures and Tables

**Table 1:** Baseline Characteristics of Participants by Group (N = 182).

Characteristic	Frequency (N = 182)	0 Dose (n = 56, 30.8%)	1 Dose (n = 70, 38.5%)	2 Doses (n = 56, 30.8%)	*p*-Value
	n (% of Total)	n (% of Total)	n (% of Total)	n (% of Total)	
Sex					
Male	68 (37.4)	11 (19.6)	29 (41.4)	28 (50.0)	0.003
Female	114 (62.6)	45 (80.4)	41 (58.6)	28 (50.0)	
Age					
15–25	56 (30.8)	26 (46.4)	21 (30.0)	9 (16.1)	0.002
26–45	64 (35.2)	19 (33.9)	27 (38.6)	18 (32.1)	
46+	62 (34.1)	11 (19.6)	22 (31.4)	29 (51.8)	
Education					
None	31 (17.0)	14 (25.0)	13 (18.6)	4 (7.1)	0.141
Primary	104 (57.1)	28 (50.0)	41 (58.6)	35 (62.5)	
Secondary	47 (25.8)	14 (25.0)	16 (22.9)	17 (30.4)	
Occupation					
Unemployed	127 (69.8)	35 (62.5)	50 (71.4)	42 (75.0)	0.313
Informal sector (self-employed)	33 (18.1)	10 (17.9)	14 (20.0)	9 (16.1)	
Formal sector (office worker)	22 (12.1)	11 (19.6)	6 (8.6)	5 (8.9)	
Floor Material					
Cement	15 (8.2)	4 (7.1)	4 (5.7)	7 (12.5)	0.364
Mud	167 (91.8)	52 (92.9)	66 (94.3)	49 (87.5)	
Source of Drinking Water					
Unimproved ^1^	97 (53.3)	31 (55.4)	40 (57.1)	26 (46.4)	0.455
Improved ^2^	85 (46.7)	25 (44.6)	30 (42.9)	30 (53.6)	
Type of Toilet facility					
Unimproved ^3^	119 (65.4)	39 (69.6)	48 (68.6)	32 (57.1)	0.295
Improved ^4^	63 (34.6)	17 (30.4)	22 (31.4)	24 (42.9)	
HIV Status					
Negative	142 (78.0)	38 (67.9)	58 (82.9)	46 (82.1)	0.087
Positive	40 (22.0)	18 (32.1)	12 (17.1)	10 (17.9)	

*p*-values based on Chi^2, 1^ Unprotected well/pond/canal. ^2^ Piped/borehole/tube well from home or community. ^3^ Open pit latrine. ^4^ Covered pit latrine/flushing toilet.

**Table 2:** Risk of Seroconverting (Ogawa).

Group	Frequency (N = 182)	Seroconverted	*p*-Value	Risk Ratio	*p*-Value	* Adjusted Risk Ratio	*p*-Value
	n (% of total)	n (% of row total)		RR (95% CI)		RR (95% CI)	
Day 15							
0 Dose	56 (30.77)	34 (60.7)	ref	ref		ref	
1 Dose	70 (38.46)	35 (50.0)	0.230	0.82 (0.60, 1.13)	0.227	0.69 (0.50, 0.93)	0.017
2 Doses	56 (30.77)	30 (53.6)	0.445	0.88 (0.64, 1.22)	0.446	0.66 (0.48, 0.93)	0.017
Day 30							
0 Dose	56 (30.77)	30 (53.6)	ref	ref		ref	
1 Dose	70 (38.46)	32 (45.7)	0.381	0.85 (0.60, 1.21)	0.379	0.96 (0.67, 1.37)	0.813
2 Doses	56 (30.77)	32 (57.1)	0.704	1.07 (0.76, 1.49)	0.704	1.26 (0.86, 1.84)	0.237

* Estimates were adjusted for age and sex.

## Data Availability

The data set cannot be shared publicly because it contains human research participant data; however, it can be made available to any interested researchers upon request. The Centre for Infectious Disease Research in Zambia (CIDRZ) Ethics and Compliance Committee is responsible for approving such requests. To request data access, one must write to the Secretary to the Committee/Head of Research Operations through this email address: info@cidrz.org, mentioning the intended use for the data, contact information, a research project title, and a description of the analysis being proposed, as well as the format it is expected. The requested data should only be used for the purposes related to the original research or study. The CIDRZ Ethics and Compliance Committee will normally review all data requests within 48–72 h (Monday–Friday) and provide notification if access has been granted or if additional project information is needed.

## Supplementary Materials

The following supporting information can be downloaded at: https://www.mdpi.com/article/10.3390/vaccines12040390/s1, Figure S1: Vibriocidal geometric mean antibody titres to Ogawa in participants with high (positive) or lower (negative) baseline IgG antibodies across treatment arms; Figure S2: Vibriocidal geometric mean antibody titres to Inaba in participants with high (positive) or lower (negative) baseline IgG antibodies across treatment arms; Figure S3: Vibriocidal geometric mean antibody titres to Ogawa in participants with high (positive) or lower (negative) baseline IgA antibodies across treatment arms; Figure S4: Vibriocidal geometric mean antibody titres to Inaba in participants with high (positive) or lower (negative) baseline IgA antibodies across treatment arms; Figure S5: Shows vibriocidal geometric mean antibody titres to serotype Inaba in participants with high (positive) or lower (negative) baseline IgM antibodies across treatment arms; Figure S6: Shows vibriocidal geometric mean antibody titres to Ogawa in participants with high (positive) or lower (negative) baseline IgM antibodies across treatment arms; Figure S7: Ogawa titers kinetics by HIV status. Emerald (HIV-), Red (HIV+); Figure S8: Inaba titers kinetics by HIV status. Emerald (HIV-), Red (HIV+).
