# Supplementary material for: Seroconversion and Kinetics of Vibriocidal Antibodies during the First 90 Days of Re-Vaccination with Oral Cholera Vaccine in an Endemic Population
Source: Vaccines (Basel). 2024 Apr 8;12(4):390. doi: 10.3390/vaccines12040390 (PMC11055093; doi:10.3390/vaccines12040390)
Supplement: Supplementary file 1 [file vaccines-12-00390-s001.zip › vaccines-2885472-supplementary.pdf]

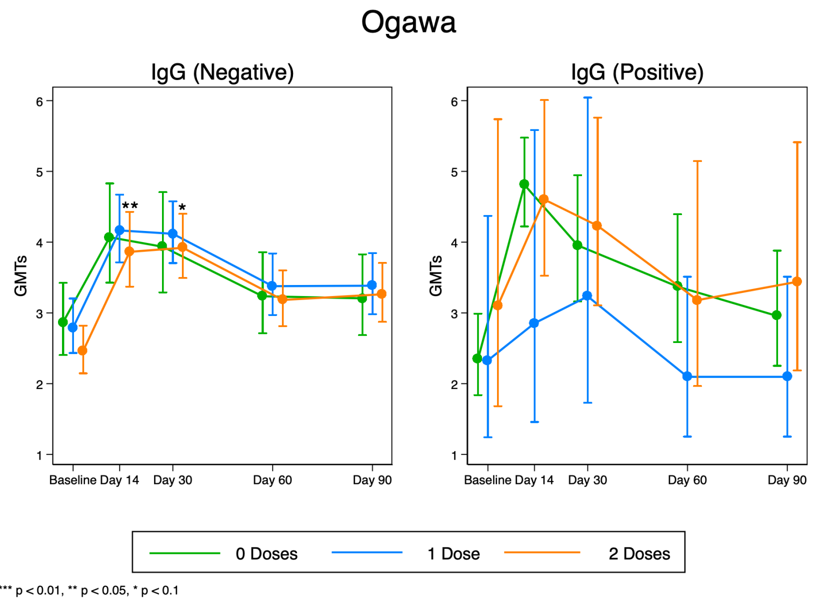

Figure S1. Shows vibriocidal geometric mean antibody titres to Ogawa in participants with high (positive) or lower (negative) baseline IgG antibodies across treatment arms.

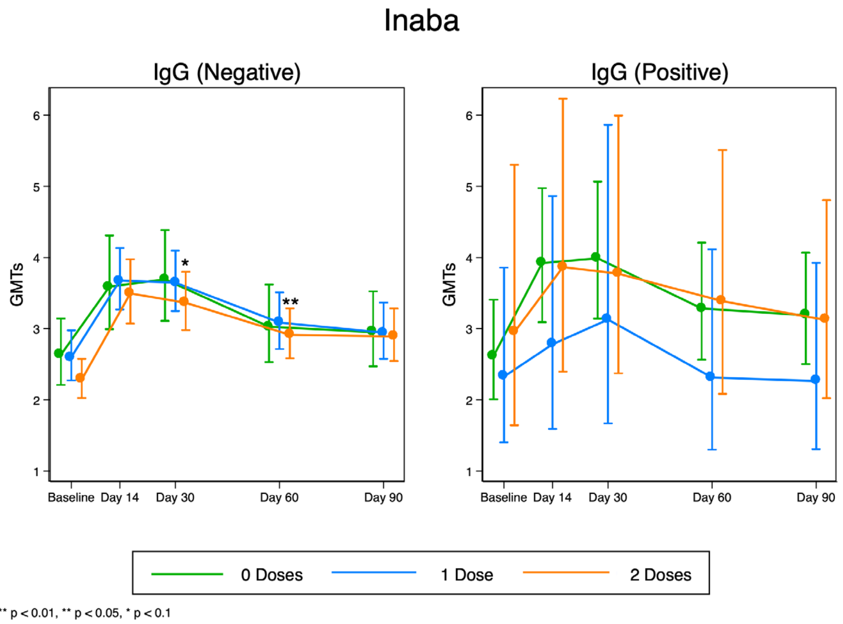

Figure S2. Shows vibriocidal geometric mean antibody titres to Inaba in participants with high (positive) or lower (negative) baseline IgG antibodies across treatment arms.

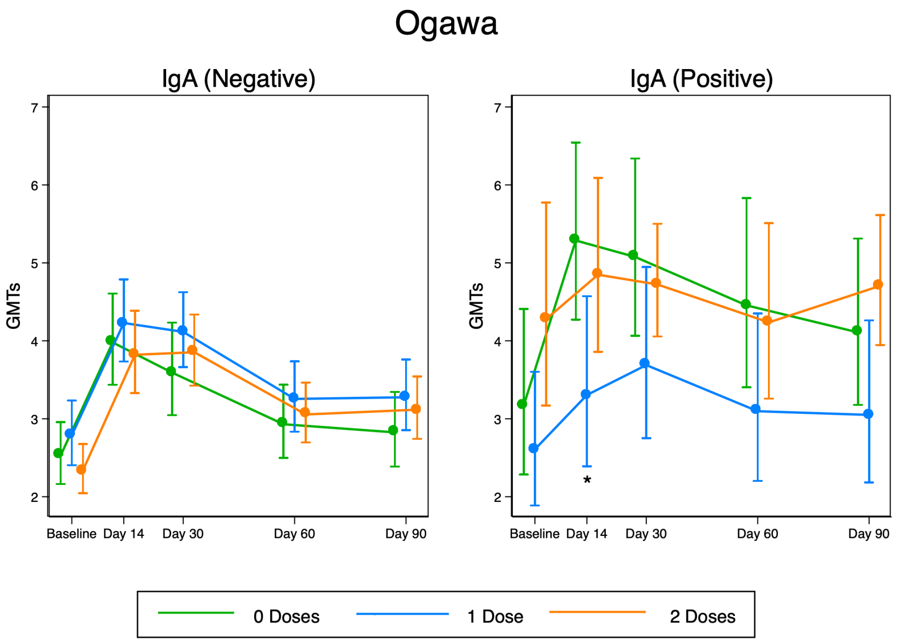

*** p < 0.01, ** p < 0.05, * p < 0.1

Figure S3. Shows vibriocidal geometric mean antibody titres to Ogawa in participants with high (positive) or lower (negative) baseline IgA antibodies across treatment arms.

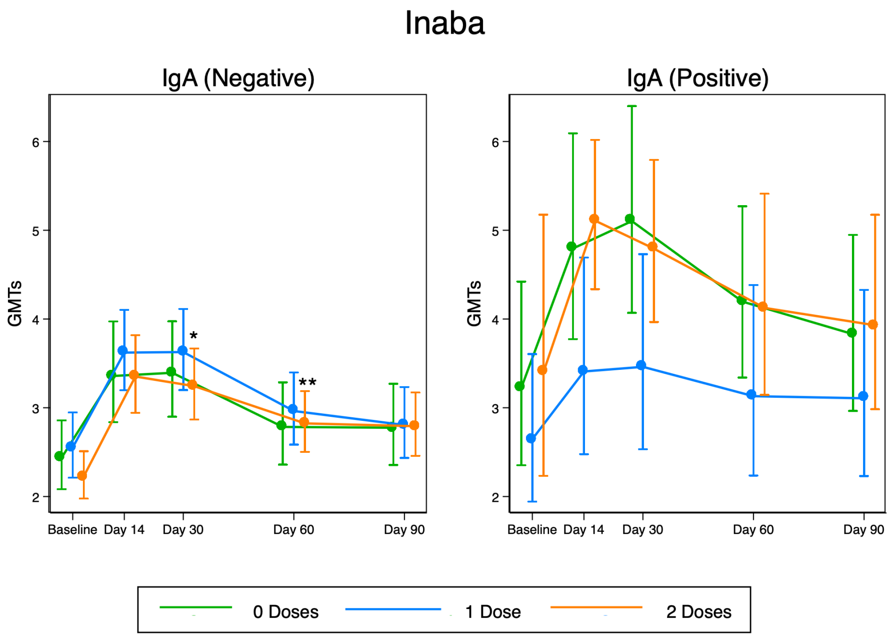

*** p < 0.01, ** p < 0.05, * p < 0.1

Figure S4. Shows vibriocidal geometric mean antibody titres to Inaba in participants with high (positive) or lower (negative) baseline IgA antibodies across treatment arms.

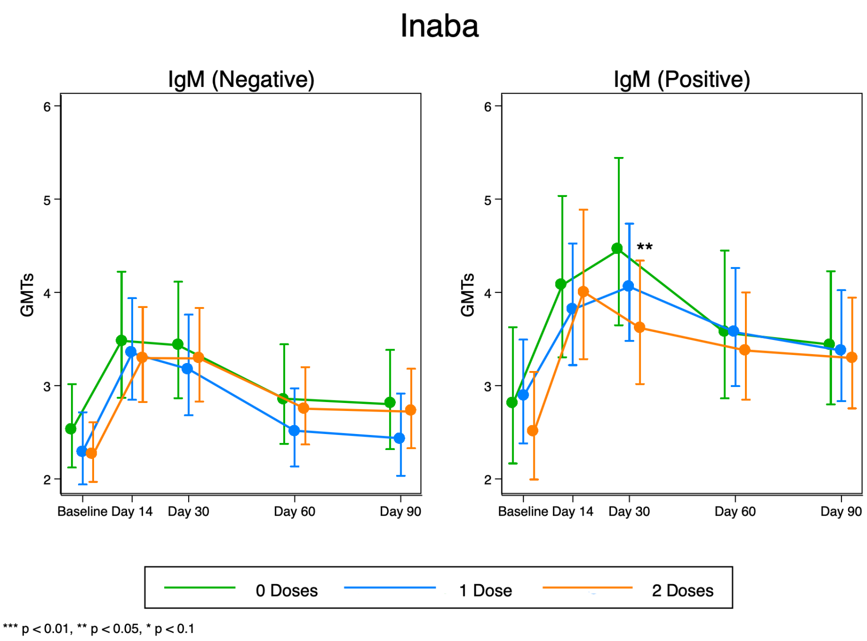

Figure S5. Shows vibriocidal geometric mean antibody titres to serotype Inaba in participants with high (positive) or lower (negative) baseline IgM antibodies across treatment arms.

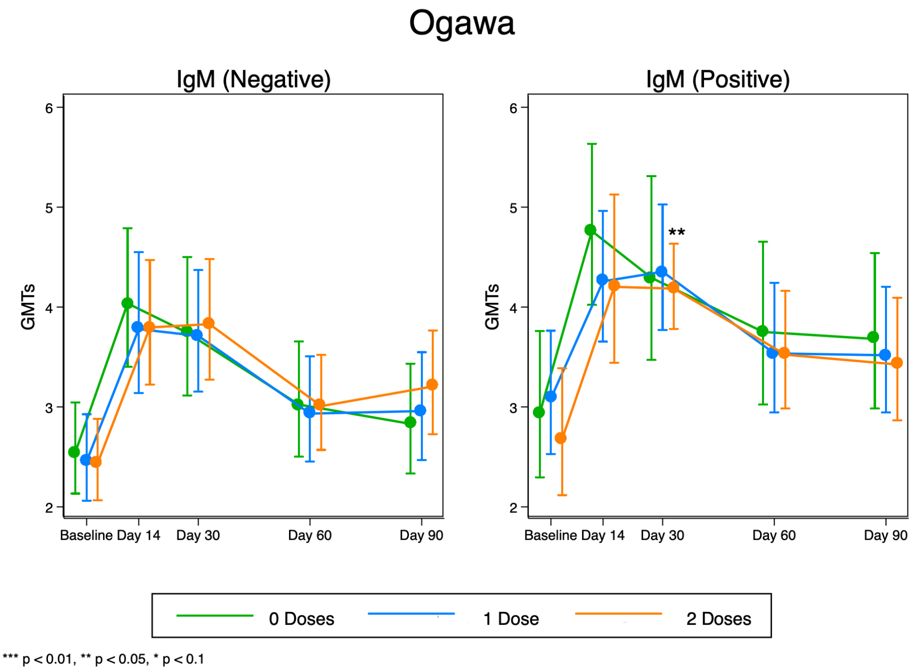

Figure S6. Shows vibriocidal geometric mean antibody titres to Ogawa in participants with high (positive) or lower (negative) baseline IgM antibodies across treatment arms.

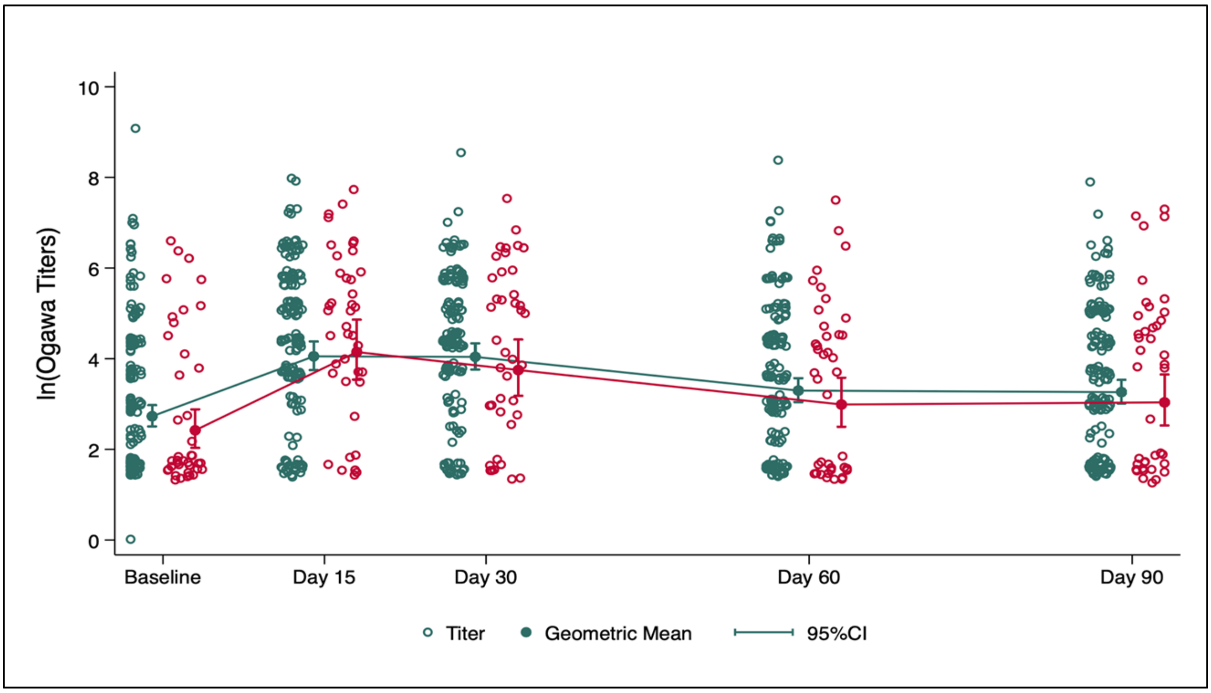

Figure S7. Ogawa titers kinetics by HIV status. Emerald (HIV-), Red (HIV+).

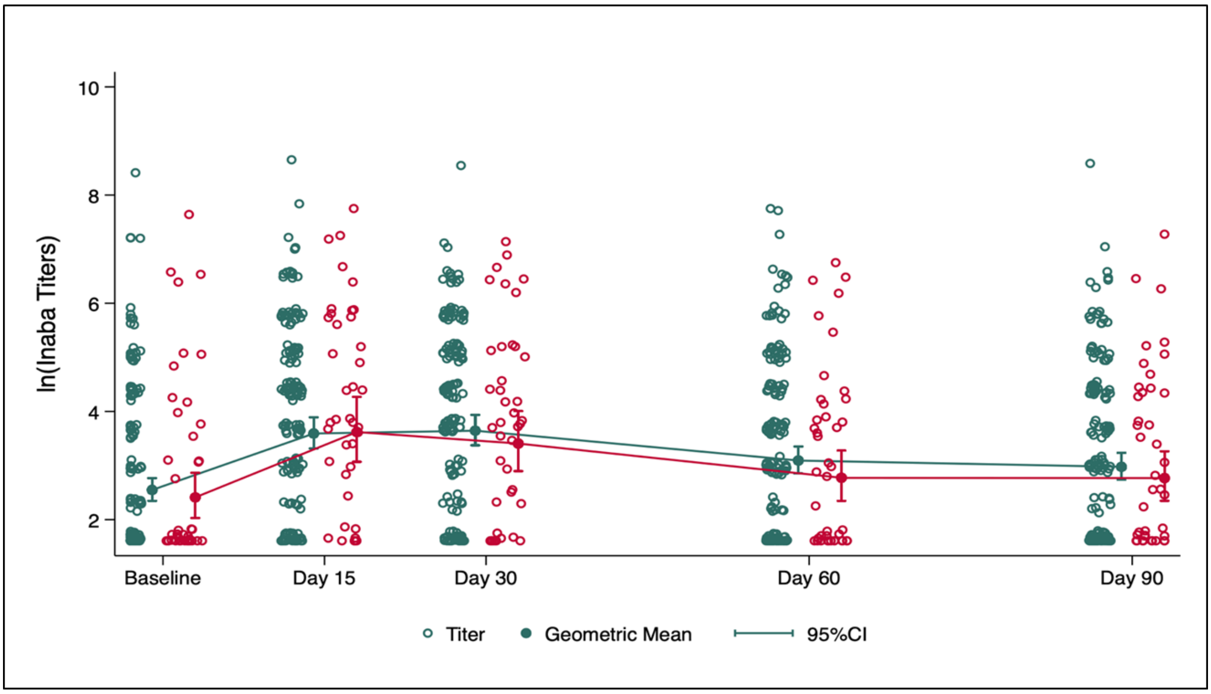

Figure S8. Inaba titers kinetics by HIV status. Emerald (HIV-), Red (HIV+).
